# Low linolenic and linoleic acid consumption are associated with chronic kidney disease in patients with type 2 diabetes

**DOI:** 10.1371/journal.pone.0195249

**Published:** 2018-08-09

**Authors:** Ana Luiza Teixeira dos Santos, Camila Kummel Duarte, Manoella Santos, Maira Zoldan, Jussara Carnevalle Almeida, Jorge Luiz Gross, Mirela Jobim Azevedo, Alice Hinda Lichtenstein, Themis Zelmanovitz

**Affiliations:** 1 Medical Sciences Post-Graduate Program: Endocrinology, School of Medicine, Universidade Federal do Rio Grande do Sul, Porto Alegre, Brazil; 2 Endocrine Division, Hospital de Clínicas de Porto Alegre, Universidade Federal do Rio Grande do Sul, Porto Alegre, Brazil; 3 Cardiovascular Nutrition Laboratory, Jean Mayer USDA Human Nutrition Research Center, Tufts University, Boston, MA, United States of America; The Pennsylvania State University, UNITED STATES

## Abstract

**Aim:**

This cross-sectional study aimed to assess the association of the fat content in the diet with Diabetic Kidney Disease (DKD) in patients with type 2 diabetes.

**Methodology:**

Patients from the Diabetes research clinic at Hospital de Clínicas de Porto Alegre (Brazil) were consecutively recruited. The inclusion criterion was the diagnosis of type 2 diabetes. The exclusion criteria were as follows: body mass index >40 kg/m^2^, heart failure, gastroparesis, diabetic diarrhea, dietary counseling by a registered dietitian during the previous 12 months, and inability to perform the weighed diet records (WDR). The dietary fatty acids (saturated, monounsaturated and polyunsaturated) consumption was estimated by 3-day WDR. Compliance with the WDR technique was assessed by comparison of protein intake estimated from the 3-day WDR and from the 24-h urinary nitrogen output performed on the third day of the WDR period. The presence of DKD was defined as urinary albumin excretion (UAE) ≥ 30 mg / 24 h or/and glomerular filtration rate (eGFR) <60 ml/min/1.73 m^2^. Urinary albumin was measured twice and eGFR was estimated by using the CKD-EPI equation.

**Results:**

A total of 366 patients were evaluated; of these, 33% (n = 121) had DKD. Multivariate analysis showed that the intake of linolenic acid was negatively associated with DKD (OR = 0.57; 95% CI 0.35–0.93; P = 0.024), adjusted for gender, smoking, cardiovascular disease, ACE inhibitors and/or angiotensin receptor blocker use, systolic blood pressure, fasting plasma glucose and HDL cholesterol. In a separate model, similar results were observed for linoleic acid, adjusting to the same co-variables (OR = 0.95; 95% CI 0.91–0.99; P = 0.006).

**Conclusion:**

The lower intake of polyunsaturated fatty acids, especially linolenic and linoleic acid, is associated with chronic kidney disease in patients with type 2 diabetes.

## Introduction

Chronic kidney disease is a major microvascular complication of diabetes and affects about one third of the patients [1]. Although aggressive and multifactorial control of traditional risk factors may attenuate the progression of this complication [2], it is still an important global public health problem [3]. Hence, this reinforces the importance of identifying additional risk factors that might be associated with the development of Diabetic Kidney Disease (DKD) and develop strategies to mitigate their effects. Some dietary components are directly related to renal function and may play a role in the development and progression of DKD.

For a long time, there have been many reports about the role of the dietary fat content in the development of chronic kidney disease [4], both in people with and without diabetes. Some observational studies have reported a positive association between increasing values of albuminuria and dietary saturated fatty acids (SFA) [5,6], as well as an inverse association with polyunsaturated FA (PUFA) [5], both in patients with type 1 and type 2 diabetes. Previous interventional trials in patients with type 2 diabetes and DKD also demonstrated a reduction of albuminuria after experimental diets with higher PUFA content (e.g., chicken-based in place of red meat; or soy protein enriched diets in place of animal protein) [7,8]. In regard to supplementation of n-3 PUFA, a beneficial effect on the reduction of albuminuria was observed in one meta-analysis of clinical trials [9], but not in others, especially when patients with diabetes were separately analyzed [10]. However, these findings are not enough to support dietary recommendations. More clinical research about this issue has been suggested in order to establish evidence-based specific dietary recommendations.

Many of the studies emphasize the possible association between dietary fat content and renal dysfunction assessed by albuminuria, and a few of them by glomerular filtration rate (GFR). In non-diabetic patients, a higher consumption of PUFA was associated with a lower risk for the presence of a GFR <60 ml/min/1.73 m^2^ [11,12], but it was not observed in other studies [13,14]. In a longitudinal study conducted with patients with type 2 diabetes, it was observed that high erythrocyte PUFAs, especially n-3 or n-3/n-6 PUFA ratio, were independently associated with a lower risk of renal function decline [15]. Therefore, the aim of the present study is to evaluate the association of dietary fat composition with DKD in patients with type 2 diabetes, based on the presence of albuminuria and/or GFR <60 ml/min/1.73 m^2^.

## Materials and methods

### Study population

In this cross-sectional study, patients from the Diabetes research clinic at Hospital de Clínicas de Porto Alegre (Rio Grande do Sul, Brazil) were consecutively recruited based on the following inclusion criterion: diagnosis of type 2 diabetes, defined as patients over 30 years of age at the onset of diabetes, no previous episodes of ketoacidosis or documented ketonuria and, if insulin users, the treatment with insulin began only 5 years after diagnosis [16]. The exclusion criteria were as follows: body mass index (BMI) >40 kg/m^2^, heart failure, gastroparesis, diabetic diarrhea, dietary counseling by a registered dietitian during the previous 12 months, and inability to perform the weighed diet records (WDR). The study was approved by the Hospital Ethics Committee (“Comitê de Ética em Pesquisa do Hospital de Clínicas de Porto Alegre”—CEP/HCPA) and all patients signed a written informed consent form. The protocol and the present manuscript followed the STROBE guideline.

All patients underwent a standardized clinical, nutritional and laboratory examination. The clinical assessment emphasized the evaluation for diabetic chronic complications. Mean blood pressure was calculated based on two separate measures using a digital sphygmomanometer (OMRON® Automatic Blood Pressure Monitor, Model HEM-705CP, Vernon Hills, Illinois 60061). Hypertension was defined as blood pressure ≥ 140/ 90 mmHg or use of antihypertensive drugs on at least two separate occasions. Patients were classified as “nonsmoker” or “smoker” (smoker in the past or currently a smoker).

### Dietary assessment

**Fig 1:**
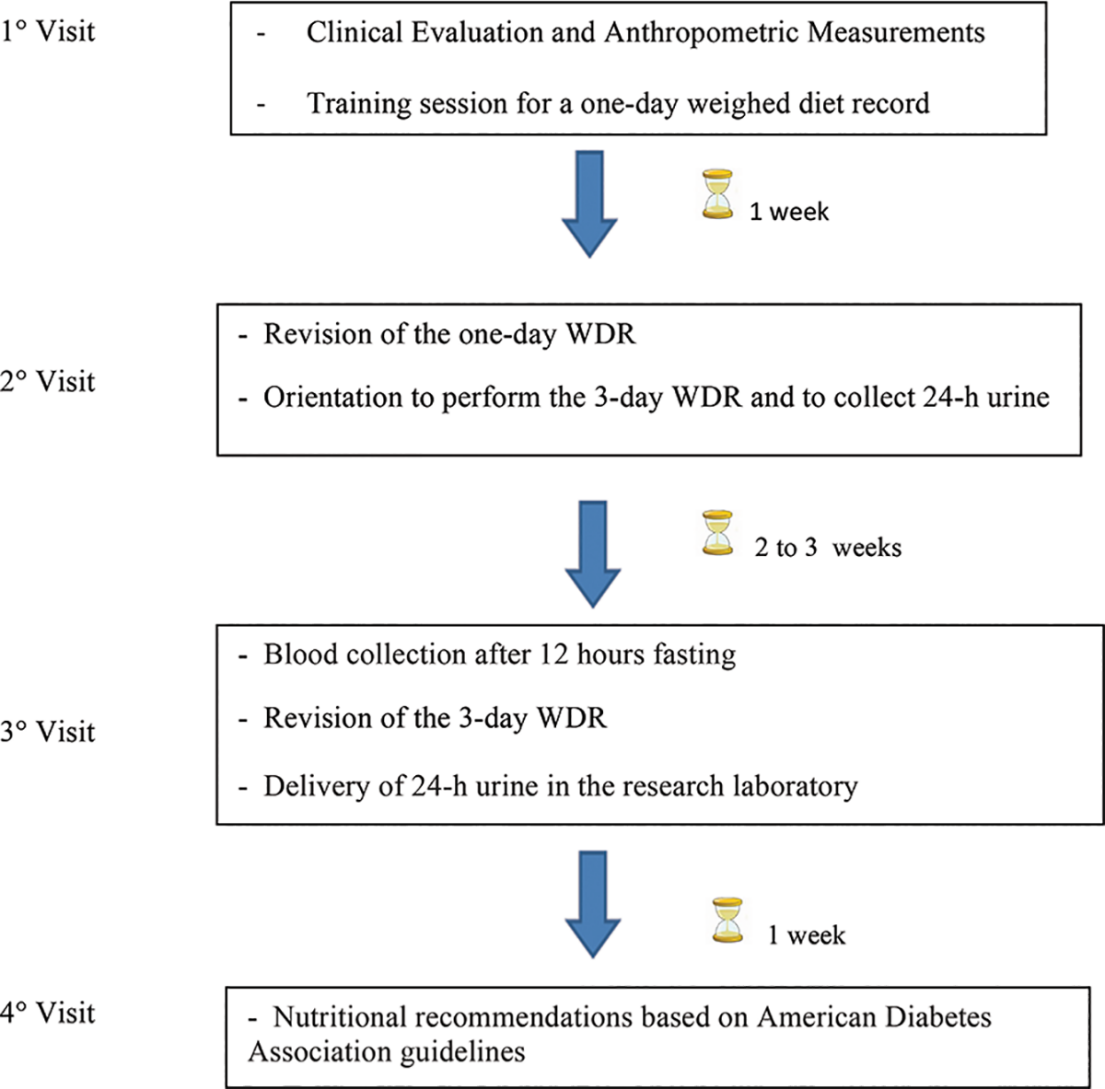
Diagram of the study design.

Diet was assessed using a 3-day WDR technique (two nonconsecutive weekdays and one day of the weekend). Patients were issued commercial scales (1-125g) and measuring cups (25–250 mL; Pyrex), and given a detailed explanation of the procedures by a trained, registered dietitian. The 3-day WDR was performed over two to three weeks and before nutritional counseling was provided for all patients **(Fig 1).**

Compliance with the WDR technique was assessed by comparison of protein intake estimated from the 3-day WDR (PI-WDR) with protein intake estimated from the 24-h urinary nitrogen output (PI-N) performed on the third day of the WDR period [17]. Patient compliance with the WDR protocol is established if the PI-WDR/ PI-N ratio remained between 0.79 and 1.26 [17].

In the 3-day WDR technique, it was intensely focused on estimating oil intake (besides individual oil used at table—in salads for example), also considering the number of people that usually share meals where oil was used to prepare the foods.

The analysis of dietary nutrients from the 3-day WDRs was performed using the Nutribase Clinical Nutritional Manager software (version 7.14, 2007; Cybersoft, Phoenix, AZ). Nutritional data for frequently consumed foods was updated, if necessary, and/or complemented with data obtained from local manufacturers. The mean values of each nutrient consumed during the 3-day WDR were calculated.

The intake of 40 individual dietary fatty acids was analyzed by Nutribase software: 14 SFA, 11 monounsaturated (MUFA) and 15 PUFA. In this software, the sum of the individual FA of each group was also determined. In the present study, the analysis of dietary fatty acid composition focused on the consumption of each group of FA and on the median intake of the most consumed individual dietary fatty acids.

### Renal function evaluation

The renal function was evaluated by serum creatinine and 24-h urinary albumin excretion (UAE) [18]. We calculated the mean value of two measurements (within a period of 3 to 6 months) to establish persistent albuminuria (≥ 30 mg/24-h) [18]. Albuminuria was considered moderately increased when it was between 30 and 299 mg/24-h; and severely increased when it was ≥ 300 mg/24-h.

GFR was estimated by using the CKD-EPI equation: eGFR = 141 x min (Scr/k,1)^a^ x max (Scr/k,1)^-1.209^ x 0.993^Age^ x 1.018 (if female) x 1.159 (if black), where Scr is serum creatinine, k is 0.7 for females and 0.9 for males, ^a^ is -0.329 for females and -0.411 for males, min indicates minimum of Scr/k or 1, and max indicates maximum of Scr/k or 1 [19]. The definition of DKD was based on the presence of UAE ≥ 30 mg /24 h and/or eGFR <60 ml/min/1.73 m^2^ for 3 months or more [18].

### Laboratory analyses

Glycemic control was evaluated by serum glucose (glucose-peroxidase colorimetric enzymatic method) and glycated hemoglobin (high precision chromatography). The lipid profile consisted of the measurement of total cholesterol and triglycerides using colorimetric assay, HDL cholesterol (direct enzymatic method), and LDL cholesterol (calculated using the Friedewald formula). Serum creatinine was measured by the Jaffe method and urea by kinetic UV assay. Urinary albumin excretion was evaluated by the immunoturbidimetry technique (MicroAlb Sera-Pak Immuno Microalbuminuria, Bayer, Tarrytown, NY) [20].

### Statistical analysis

Student t test, Mann-Whitney U test, and the Exact Fisher or Chi-Square tests were applied. Multivariable logistic regression models were used to assess the associations of DKD (dependent variable) with dietary fats (independent variables). Different models were generated for each nutrient and the other independent variables were selected based on their significance (P < 0.10) at univariate analysis or according to their biological relevance.

Variables with a non-Gaussian distribution (serum triglycerides, UAE, linolenic acid and *trans* fatty acids intake) were log-transformed before analysis. Data were expressed as mean ± standard deviation or as median (minimum-maximum) unless otherwise stated. The level of significance adopted was 5%. Software SPSS 18.0 (SPSS®, Chicago, IL) was used for the analysis.

The sample size necessary for the study was calculated based on SFA and PUFA intake of type 1 and type 2 diabetic patients with and without DKD from two previous studies, using the WINPEPI V11.65 software. In the study of Riley et al [21], a difference of 1.2% of energy of SFA intake was observed between normo- and microalbuminuric patients. Considering a statistical power of 80% and a level of significance of 5%, the estimated number of patients necessary would be 311 (84 with and 227 without DKD). The second study by Almeida et al [22] reported a difference of 1.1% of energy on PUFA intake between type 2 diabetic patients with and without microalbuminuria. Based on this result, the estimated number of patients necessary would be 297 (99 with and 198 without DKD).

## Results

### Patient characteristics

A total of 366 patients (177 male) underwent clinical, laboratory, and nutritional evaluation. Thirty-three percent of the patients presented DKD **(Table 1)**. Of these, 68% had a moderately elevated UAE (30-300mg/24-h) and 22% had a markedly elevated UAE (>300mg/24-h). The great majority of patients were in Stage 1 and 2 of the Chronic Kidney Disease classification of the National Kidney Foundation and no patient was in Stage 5 of CKD-EPI **(Table 2).**

**Table 1:** Clinical and laboratory characteristics of patients with type 2 diabetes divided according to the presence or not of diabetic kidney disease.

	Without DKD (n = 245)	With DKD (n = 121)	P
**Age (years)**	61 ± 10	60 ± 10	0.21
**Male (%)**	44	58	0.01
**Duration of diabetes (years)**	12 ± 8	13 ± 8	0.94
**Ethnicity: White**	85%	86%	0.94
**Hypertension**^**a**^	74%	90%	<0.001
**Cardiovascular Disease**	20.4%	35.2%	0.003
**Smoking**	48%	63%	0.01
**BMI (kg/m**^**2**^**)**	28.5 ± 4.4	28.7 ± 4.2	0.64
**Waist Circumference (cm)**			
**Men**	100 ± 10	102 ± 12	0.13
**Women**	98 ± 11	100 ± 10	0.42
**Systolic Blood Pressure (mmHg)**	137 ± 20	141 ± 22	0.06
**Diastolic Blood Pressure (mmHg)**	79 ± 11	82 ± 12	0.12
**RAAS agents**	56%	69%	0.02
**Hypolipidemic agents**	35%	27%	0.12
**Fasting plasma glucose (mg/dL)**	147 ± 51	160 ± 66	0.04
**Glycated hemoglobin (%)**	7.4 ± 1.5	7.7 ± 1.6	0.11
**Total cholesterol (mg/dL)**	200 ± 41	204 ± 45	0.48
**HDL cholesterol (mg/dL)**	51 ± 13	48 ± 11	0.02
**LDL cholesterol (mg/dL)**	121 ± 34	122 ± 41	0.78
**Triglycerides (mg/dL)**	135 (25–421)	144 (49–573)	0.18

BMI: body mass index; RAAS agents: renin angiotensin aldosterone system agents (angiotensin-converting enzyme inhibitors or angiotensin receptor blockers).

^a^Hypertension was defined as blood pressure ≥ 140/ 90 mmHg or use of antihypertensive drugs on at least two separate occasions. Data are expressed as mean ± SD, median (95% confidence interval), or number of patients (percentage) with the characteristic.

**Table 2:** Renal function status of patients with type 2 diabetes divided according to the presence or not of diabetic kidney disease.

	Without DKD (n = 245)	With DKD (n = 121)
**eGFR (ml/min/1.73 m**^**-2**^**)**	94.2 ± 16.0	93.8 ± 24.9
**Stage 1 [n (%)]**	155 (63.3)	77 (63.6)
**Stage 2 [n (%)]**	90 (36.7)	25 (20.7)
**Stage 3a [n (%)]**	-	15 (12.4)
**Stage 3b [n (%)]**	-	2 (2.5)
**Stage 4 [n (%)]**	-	1 (0.8)
**Albuminuria (mg/24-h)**	3.6 (0–28.7)	93.9 (3–3570.0)
**Normal [n (%)]**	245 (100)	12 (9.9)
**Moderately increased [n (%)]**	-	82 (67.8)
**Severely increased [n (%)]**	-	27 (22.3)

eGFR: estimated glomerular filtration rate.

According to albuminuria values and/or eGFR, the patients were divided into two groups: with and without DKD. In the patients with DKD, there were a higher proportion of males, as well as individuals with a smoking habit (**Table 1**). As expected, hypertension and cardiovascular disease were more frequent in patients with DKD. Patients with DKD more frequently used the renin angiotensin aldosterone system agents (angiotensin-converting enzyme inhibitors or angiotensin receptor blockers). These patients had worse glycemic control and lower serum levels of HDL cholesterol than patients without DKD. No patient presented BMI < 18.5 kg/m^2^ in both groups, suggesting malnourishment.

### Analyses of dietary nutrients and diabetic kidney disease

Participants with DKD reported a higher intake of dietary protein compared to those without DKD **(Table 3)**. However, when dietary protein intake was evaluated as g/kg of body weight, no significant difference was observed between the patients with (1.17 ± 0.34 g/kg/) and without DKD (1.17 ± 0.33 g/kg; P = 0.92).

**Table 3:** Daily dietary intake of patients with type 2 diabetes divided according to the presence or not of diabetic kidney disease.

	Without DKD (n = 245)	With DKD (n = 122)	P
**Energy (kcal)**	1830 ± 503	1806 ± 494	0.66
**Carbohydrates**			
g/day	213.9 ± 63.3	213.8 ± 70.5	0.98
% of energy	46.9 ± 6.5	47.3 ± 7.9	0.70
**Proteins**			
g/day	86.1 ± 27.1	89.8 ± 28.1	0.23
% of energy	18.9 ± 3.2	20.1 ± 4.3	0.01
**Lipids**			
g/day	69.8 ± 24.8	65.8 ± 24.9	0.15
% of energy	34 ± 6.9	32.6 ± 7.9	0.07
**Saturated FA**			
% of energy	9.6 ± 2.5	9.3 ± 2.9	0.28
% of total fat	28.4 ± 6.2	28.4 ± 5.5	0.99
**Monounsaturated FA**			
% of energy	11.4 ± 2.7	11.5 ± 2.9	0.57
% of total fat	33.6 ± 5.2	35.7 ± 5.1	<0.01
**Polyunsaturated FA**			
% of energy	9.98 ± 3.4	8.77 ± 3.7	<0.01
% of total fat	29.2 ± 7.6	26.6 ± 7.2	<0.01
**P/S Ratio**	1.1 ± 0.5	1.0 ± 0.5	0.07
**Cholesterol (mg)**	207 ± 107	220 ± 98	0.27
***Trans* FA**			
% of energy	1.01 (0–5.67)	1.07 (0–3.75)	0.61
% of total fat	3.06 (0–14.4)	3.3 (0–12.7)	0.34

DKD: Diabetic Kidney Disease FA: fatty acid; P/S ratio: polyunsaturated/saturated ratio. Data are expressed as mean and standard deviation, or median and interquartile range.

Regarding dietary fat, participants with DKD reported a lower total PUFA intake **(Table 3)**. Analyzing the individual dietary PUFA, subjects with DKD presented lower linolenic acid and linoleic acid intake **(Table 4).** The group of patients with DKD also reported a higher intake of dietary monounsaturated fatty acids, but only when it was expressed as percent of total fat. When dietary monounsaturated fatty acid was expressed as g/day (without DKD: 23.4 ± 9.1 g/day; with DKD: 23.4 ± 9.2 g/day; P = 0.98) or as % of energy, no difference was observed between the groups **(Table 3)**. Moreover, no association was observed for evaluated individual dietary SFA and MUFA intake.

**Table 4:** Individual dietary polyunsaturated fatty acids of patients with type 2 diabetes divided according to the presence or not of diabetic kidney disease.

	Without DKD (n = 245)	With DKD (n = 122)	P
**Linoleic FA (18:2n-6)**			
**g/day**	17.8 ± 7.5	15.5 ± 8.9	0.01
**% of total fat**	25.7 ± 6.8	23.3 ± 6.9	<0.01
**Arachidonic FA (20:4n-6)**			
**g/day**	0.1 (0.02–0.5)	0.1 (0–0.32)	0.26
**% of total fat**	0.2 (0.03–0.9)	0.2 (0–0.54)	0.06
**Linolenic FA (18:3n-3)**			
**g/day**	1.9 (0.3–5.9)	1.5 (0–9.2)	0.01
**% of total fat**	2.9 (0.6–7.7)	2.6 (0–7.3)	0.08
**Eicosapentaenoic FA (20:5n-3)**			
**g/day**	0.01 (0–0.32)	0.01 (0–0.26)	0.53
**% of total fat**	0.01 (0–0.64)	0.01 (0–0.38)	0.31
**Docosahexaenoic FA (22:6n-3)**			
**g/day**	0.02 (0–0.97)	0.02 (0–0.76)	0.41
**% of total fat**	0.03 (0–1.9)	0.04 (0–1.12)	0.16

Data are expressed as mean and standard deviation, or median and interquartile range.

Multivariate analysis showed that the total PUFA consumption was negatively associated with DKD (OR = 0.95; 95% CI: 0.92–0.986; P = 0.005), adjusted for gender, smoking, cardiovascular disease, renin angiotensin aldosterone system agents use, systolic blood pressure, fasting plasma glucose, HDL cholesterol and total energy intake **(Table 5)**. When individual FA were separately analyzed in other models, similar results were observed both for linoleic acid (OR = 0.95; 95% CI: 0.91–0.985; P = 0.006) and for linolenic acid (OR = 0.57; 95% CI: 0.35–0.93; P = 0.024), adjusting to the same co-variables. No association was observed for other evaluated PUFA: eicosapentaenoic acid (EPA), docosahexaenoic acid (DHA), and arachidonic acid.

**Table 5:** Logistic regression analysis—dependent variable: the presence of diabetic kidney disease based on the values of albuminuria > 30 mg/day and/or eGFR <60 ml/min/1.73 m^2^.

Nutrients ^a^	OR	IC– 95%	P
**Polyunsaturated FA (% of fatty acids)**	0.95	0.92–0.99	0.005
**Linoleic FA (18:2n-6) (% of fatty acids)**	0.95	0.91–0.99	0.006
**Linolenic FA (18:3n-3) (% of fatty acids)**	0.57	0.35–0.93	0.024

FA: fatty acid

^a^ Each nutrient was separately analyzed in models adjusted for gender, smoking, cardiovascular disease, use of Renin Angiotensin Aldosterone System agents, systolic blood pressure, fasting plasma glucose, HDL cholesterol and total energy intake.

Soybean oil was the most widely consumed vegetable oil (70%) for all patients; other oils consumed included sunflower, corn, rice or the combination of more than one type of oil. The proportion of soybean oil intake of patients with DKD was significantly lower (41%) than for patients without DKD (71%; P = 0.01). In relation to other foods sources of n-3 PUFA, only 14% of patients consumed marine foods, with no significant difference between patients with (16%) and without (15%) DKD.

When the patients were divided according to degrees of albuminuria, it was observed that the consumption of total PUFA, as well as linoleic acid, was significantly higher in the normoalbuminuric group, compared to the group with severely elevated albuminuria. When the linolenic acid intake was analyzed, both the normoalbuminuric group and those with moderately elevated albuminuria presented a higher intake of this FA, compared to the group with severely elevated albuminuria **(Table 6).**

**Table 6:** Dietary polyunsaturated fatty acids content of type 2 diabetic patients according to the degree of albuminuria.

	Normo (n = 257)	Moderately increased albuminuria (n = 82)	Severely increased albuminuria (n = 27)	P
**PUFA** **(% of energy)**	9.9 ± 3.4 ^a^	9.2 ± 4.1	8.1 ± 3.2	0.02
**Linoleic FA (% of energy)**	8.7 ± 3.0 ^a^	8.1 ± 3.8	7.1 ± 3.3	0.02
**Linolenic FA (% of energy)**	0.91 (0.18–2.42)	0.83 (0.00–3.02)	0.69 (0.01–2.12)^b^	0.03

PUFA: polyunsaturated fatty acids

^a^ Normo *vs* Severely increased albuminuria

^**b**^ Normo and moderately increased albuminuria *vs* severely increased albuminuria. Data are expressed as mean and standard deviation, or median and interquartile range.

When the patients were stratified according to the presence of eGFR values above or below 60 ml/min/1.73 m^-2^, and not considering albuminuria values, participants with lower eGFR were older (69 ± 5 vs. 60 ± 10 years; P<0.001), and more frequently have cardiovascular disease (47.4 vs. 24.2%; P = 0.03) and a smoking habit (78 vs. 52%; P = 0.03). With respect to diet, participants with eGFR levels <60 ml/min/1.73 m^2^ had a lower total total fat intake (30 vs. 34% of energy; P = 0.01). In multivariate analysis, total lipid intake (OR = 0.92; 95% CI: 0.85–0.99; P = 0.025) remained inversely associated with eGFR <60 ml/min/1.73 m^2^, adjusted for age, smoking and cardiovascular disease. No association was observed for other nutrients.

### Analyses according to tertiles of polyunsaturated fatty acids intake and diabetic kidney disease

Total and fractions of PUFA were analyzed according to the tertiles of intake. The tertiles of total PUFA intake were linearly associated with a lower proportion of patients with DKD, as follows: 1st tertile (≤ 7.66% of energy): 46%; 2nd tertile (7.67–10.50% of energy): 33%; and 3rd tertile (>10.51% of energy): 20% of DKD (P for trend < 0.01). This inverse association was confirmed by a multivariate analysis using the 1st tertile of PUFA intake as the reference (OR = 0.59, 95% CI: 0.43–0.80; P = 0.001), and adjusted for gender, smoking, ischemic heart disease, use of renin angiotensin aldosterone system agents, systolic blood pressure, fasting plasma glucose and HDL cholesterol. The same was observed for the LA and ALA as follows: LA: OR = 0.60, 95% CI: 0.44–0.82; P = 0.001; ALA: OR = 0.69, 95% CI: 0.51–0.94; P = 0.018, after adjustment for the same covariates.

**Fig 2:**
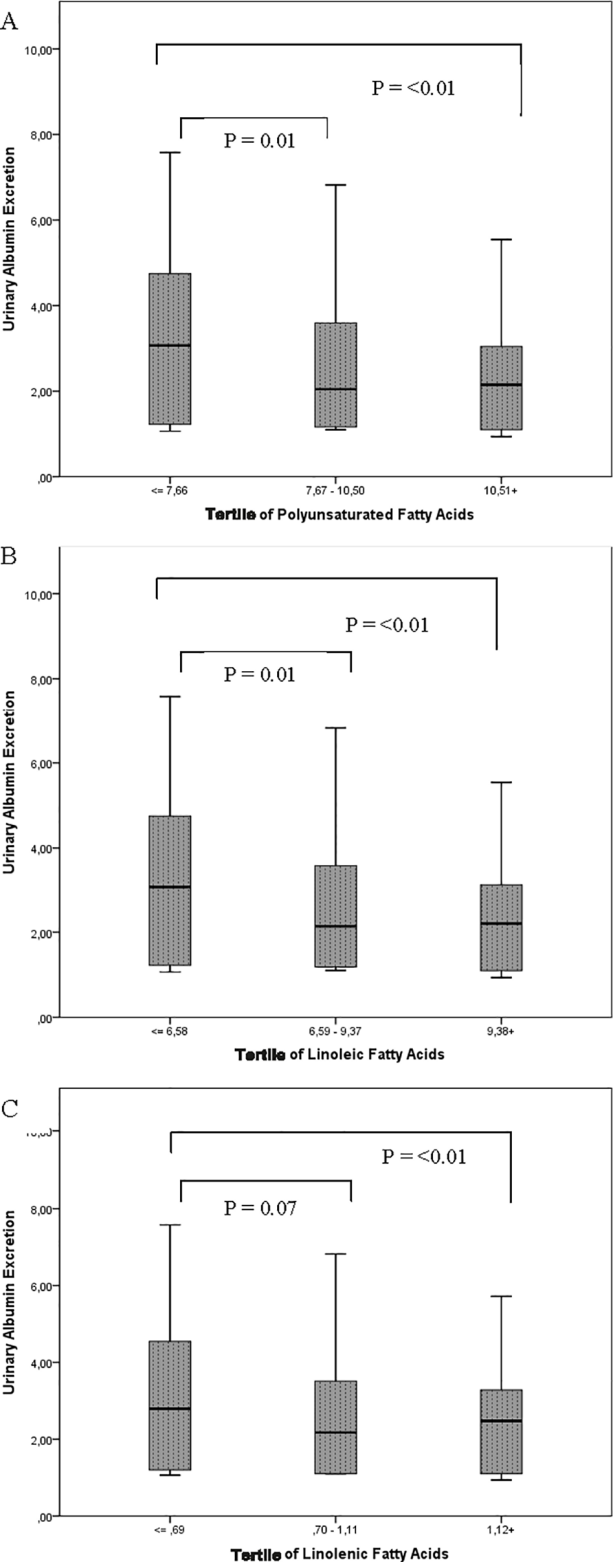
Albuminuria values in the tertiles of dietary fatty acids intake. 2A. Tertiles of dietary polyunsaturated fatty acids (% energy). 2B. Tertiles of dietary linoleic acid (% energy). 2C. Tertiles of dietary linolenic acid (% energy). Urinary Albumin Excretion is expressed log-transformed.

There was a progressive decline of albuminuria values in the tertiles of PUFA intake, for both LA and ALA **(Fig 2A, 2B and 2C)**. Albuminuria was significantly higher in the first tertile of PUFA when compared with the second and third tertile [UAE: 22 (4–116), 8 (3–37) and 9 (3–23) mg/24-h, respectively]. The same was observed for the tertiles of LA [UAE: 21 (3–116), 9 (3–37) and 9 (3–23) mg/24-h, respectively] and for the tertiles of ALA [UAE: 15 (3–94), 9 (3–35) and 12 (3–27) mg/24-h, respectively] intake.

## Discussion

The current study demonstrated a negative association of PUFA intake with DKD in patients with type 2 diabetes, even when the use of renin angiotensin aldosterone system agents and cardiovascular risk factors was taken into consideration. These data suggest diets that are higher in PUFA may have clinically beneficial effects. Moreover, our findings suggest that this association is stronger in more advanced stages of renal involvement, i.e., the patients with severely increased albuminuria presented a lower intake of these PUFA. We had previously reported that low intake of PUFA, mainly from vegetable oils, was associated with the presence of microalbuminuria in patients with type 2 diabetes, but we did not include patients with severely increased albuminuria nor evaluated GFR [22]. In a previous longitudinal study that included patients with type 1 and 2 diabetes and DKD, those patients who presented with regression of albuminuria had higher intakes of PUFA and a lower intakes of SFA. However, the investigators did not analyze individual FA [5]. Also, in clinical trials performed in type 2 diabetes patients with micro and macroalbuminuria [6,7], the short-term replacement of red meat by chicken meat, resulting in a decrease in SFA intake, resulted in a reduction in the UAE, concomitant with a higher proportion of PUFA on serum lipids [6].

Regarding the individual PUFA, the inverse association with DKD was especially significant for dietary LA and ALA. In our previous study, we also observed the lower intake of n-6 PUFA were high in patients with microalbuminuria. Likewise, this inverse association was reinforced when serum fatty acid composition, used as a biological marker of fat type intake, was evaluated in patients with type 2 diabetes [23]. In contrast, in a randomized study with type 1 diabetes patients who presented with increased albuminuria, the UAE increased by 58% after a higher intake of LA, albeit, an improvement in serum lipid profile [24]. But this study did not include patients with macroalbuminuria. In fact, more recently, Gopinath et al [12], in 2,600 healthy adults, observed a non-significant increase in the prevalence of moderate CKD with increasing consumption of n-6 PUFA. In this way, the effect of n-6 PUFA on renal disease is controversial and not well established.

Concerning the beneficial effects of dietary ALA and CKD, this subject is poorly explored and contradictory results were observed [12]. In the cohort of the Blue Mountains Eye Study, ALA intake was positively associated with CKD [12]. The authors themselves argued that this finding was not expected, and emphasize that one of the possibilities for these results is the fact that this FA, a plant n-3 PUFA, is poorly converted to EPA and DHA, which are thought to have beneficial effects on renal function. However, the ALA itself could have beneficial effects, such as, anti-inflammatory properties that may be the link between this FA and the cardiometabolic risk [25,26]. In fact, in a recent cohort study of patients with type 2 diabetes without ischemic heart disease, a high intake of PUFA, especially ALA, was protective for the development of cardiac events [27].

In our study, no significant association was observed between EPA and DHA and DKD. A renoprotective effect of these FA has been suggested by some studies conducted with diabetic patients, but not all [28]. Consumption of two or more portions of fish per week was associated with a lower risk of macroalbuminuria in individuals with diabetes [29]. Also, more recently, in a study of patients with type 1 diabetes from the DCCT study, in the cross-sectional data, an inverse association was observed between the intake of EPA and DHA and albuminuria [30]. However, in normoalbuminuric patients no relationship was observed between the intake of these FA and the incidence of albuminuria after a mean follow-up of 6.5 years [30]. Similarly, but evaluating non-diabetic participants in the Multi-Ethnic Study of Atherosclerosis, total fish consumption was not associated with albuminuria [31]. The possible explanations for these controversial findings might be the differences among the selected populations (ex: different degrees of increased albuminuria), and that some studies focused on n-3 PUFA intake, while others on dietary sources of these FA. Another possible reason is differences in the baseline dietary n-3 PUFA intake among the populations. In fact, in the present study, the mean EPA and DHA intake was low because less than 15% of our population consumes marine foods [27]. Thus, the main source of PUFA in the present study population was derived from vegetable oils, especially soybean oil.

The possible mechanisms of the beneficial effect of PUFA on kidney function may be through both blood pressure levels and improvement in lipid profile. In a cohort of American adults without hypertension at baseline, the participants in the highest quartile of n-3 PUFA intake had a significantly lower incidence of hypertension compared to those in the lowest quartile [32]. In the present study, no difference of systolic and diastolic blood pressure was observed between patients with higher and lower intake of PUFA (data not shown). Regarding the lipid profile, the group of patients with above average PUFA intake had lower levels of triglycerides when compared to the group with the lowest intake of the FA (data not shown). It is likely that lipid abnormalities may precede the development of micro- or macroalbuminuria, though this topic is not completely understood.

Some limitations of this study must be acknowledged. The study design is cross-sectional and does not allow for inference of causality. Furthermore, the small number of patients with more advanced renal involvement may have impaired the power of the study to detect an association between dietary FA and GFR decline. However, regarding the association between FA intake and the presence of DKD–defined as the presence of albuminuria and/or GFR < 60 ml/min/m2 –it is important to emphasize that the calculated power of our study considering PUFA intake was 87.4% % which was adequate. As for SFA intake, we observed a difference of only 0.3% between patients with and without DKD, which is not clinically relevant; in addition, the effect size of Cohen’s d was 0.11, suggesting that the absence of significance is true and not related to sample size. The effect size is defined as small if 0–0.2, medium if > 0.2–0.8 and large if > 0.8. Likewise, other dietary factor differences—energy, carbohydrates, lipids and monounsaturated fatty acids—were also not clinically relevant, and the effect size of Cohen’s d was, respectively, 0.05, 0.04, 0.2 and 0.06.

And, finally, linked to dietary data limitation, the measurement of a biological marker of fat type intake, like serum FA [33], would have reinforced our results. However, we used a standardized 3-day WDR, including a 24-h urinary urea measurement to confirm dietary intake estimated from records [34], which is more accurate than food frequency questionnaires. This dietary tool has been largely used to confirm dietary compliance in studies with diabetic patients [6,22,27,35]. Another aspect is that detailed attention was given to the evaluation of oil consumption, which was the main source of dietary PUFA. Furthermore, in the current study the demonstration of a significantly higher proportion of patients who consumed soybean oil in the group without (71%) than the group with DKD (41%) was independent of the amount of oil used. So this supports the inverse association between dietary intake of PUFA and DKD.

In conclusion, higher dietary intake of PUFA, especially linoleic and linolenic acids, was associated with a reduced prevalence of DKD in type 2 diabetes patients. These associations appeared to be stronger when DKD was defined by increased albuminuria and/or decreased eGFR. The availability of fish and other marine products is limited in many countries [36] and plant oils and nuts is the predominant source of PUFA in the typical Western diet. Perhaps these dietary FA are a renoprotective alternative and could be very important for public health. However, further large-scale longitudinal studies and clinical trials are needed to confirm these findings in patients with type 2 diabetes, in order to try to understand the mechanism by which this benefit occurs and thus to provide a non-drug strategy for these patients.

## Supporting information

S1 Dataset (SAV)
